# Step Rate Thresholds Associated with Moderate and Vigorous Physical Activity in Adults

**DOI:** 10.3390/ijerph15112454

**Published:** 2018-11-03

**Authors:** Myles W. O’Brien, Matthew J. Kivell, William R. Wojcik, Ghislain d’Entremont, Derek S. Kimmerly, Jonathon R. Fowles

**Affiliations:** 1 Centre of Lifestyle Studies, School of Kinesiology, Acadia University, 15 University Ave., Wolfville, NS B4P 2R6, Canada; myles.obrien@dal.ca (M.W.O.); mj.kivell@gmail.com (M.J.K.); WWojcik@dal.ca (W.R.W.); 2 Division of Kinesiology, School of Health and Human Performance, Dalhousie University, Halifax, NS B3H 4R2, Canada; ghislaindentremont@gmail.com (G.d.); Dskimmerly@dal.ca (D.S.K.)

**Keywords:** step rate, relative intensity, absolute intensity, walking

## Abstract

Adults are recommended to engage in 150 min of moderate (MPA) to vigorous (VPA) aerobic physical activity per week, with the public health message of obtaining 3000 steps in 30 min. There is a paucity of research on step rate thresholds that correspond to absolute MVPA (moderate = 3 METs, vigorous = 6 METs) with no research evaluating adult relative MVPA (moderate = 40% VO_2max,_ vigorous = 60% VO_2max_). Anthropometric differences also influence intensity-related step rate thresholds. The purpose of this study was to identify step rates across a range of walking intensities so that mathematical models incorporating anthropometric factors could be used to identify individualized MVPA step rate thresholds. Forty-three adults (25♀; age = 39.4 ± 15.2 years) completed a staged treadmill walking protocol with pedometers and indirect calorimetry: six-minutes at 2.4, 3.2, 4.0, 5.6, 6.4, 7.2 km/h. Mathematical modelling revealed absolute and relative MPA step rate thresholds of ~100 steps/minute (spm) and ~125 spm, respectively. VPA corresponded to step rates of ~133 spm and ~139 spm for absolute and relative thresholds respectively. The current public message of 3000 steps in 30 min is valid for absolute MPA. However, VPA is achieved at higher thresholds than previously reported, more than 130 spm for healthy adults.

## 1. Introduction

Physical activity is associated with a decreased risk of cardiovascular disease, cancer, and diabetes [1]. The benefits of accumulating a high daily step count, independent of intensity, is well-documented [2]. However, daily accumulated steps does not consider physical activity intensity. Given that the current national physical activity guidelines are based on physical activity done above a specific intensity and not step count, measuring intensity-related physical activity by step rate or cadence, or the effort by which those steps were taken, is particularly important. Specifically, adults are recommended to attain at least 150 min of moderate aerobic physical activity (MPA) or 75 min of vigorous aerobic physical activity (VPA) or a combination thereof, per week [3], regardless of total number of steps. Despite the health benefits of achieving these recommendations [4], the majority of the population do not meet current PA guidelines [5,6].

Objectively monitoring PA has been shown to be an effective means to increase PA levels and produce positive health outcomes such as decreased body mass index (BMI) and reduce systolic blood pressure [7]. A meta-analysis concluded that pedometer interventions typically increase PA by 2000–2500 steps per day [7]. Therefore, physical activity monitors offer a feasible, objective way to measure and monitor physical activity in public health and community settings. Recently, medical grade pedometers have been developed that allow step rate prescriptions to be tailored to individualize moderate and vigorous physical activity (MVPA) assessment [8]. This tailoring allows more accurate tracking of physical activity that meets recommended guidelines. Some of these devices (PiezoRx^®^, StepsCount, Deep River, ON, USA) are as accurate as accelerometry in quantifying adult step counts and MVPA in both laboratory and free-living conditions [8,9]. Given the health benefits of increased physical activity and the increase in physical activity resulting from monitoring, healthcare and exercise professionals may use pedometers or accelerometers to objectively assess individual physical activity levels and/or prescribe physical activity to their patients [10]. The low rates of physical activity counselling and exercise prescription among healthcare providers also demonstrates the need for tangible aids to accurately quantify patients’ physical activity levels [11,12,13].

Previous research using laboratory-based walking protocols have equated step rate and intensity-related physical activity in adults [14,15,16,17,18], with the current public message stating that moderate intensity is equal to 3000 steps in 30 min, or 100 steps per minute (spm) [15]. However, moderate intensity step rate varies among individuals of different BMI classifications, heights, and leg lengths [15,16], as individuals with shorter legs or lower body mass index will require higher step rates to achieve the same metabolic intensity as their longer leg or higher BMI counterparts. Only one study to date has examined leg length and BMI when calculating step rate thresholds for intensity-related physical activity but was limited by a small sample size (*n* = 20; age = 26.4 ± 4.6 years), and only evaluated MPA [16]. Other research either did not directly measure leg length [14,17,18], included step rate thresholds for different weight status’ [15], and/or did not evaluate step rate thresholds for VPA [14,15,16]. A recent narrative review by Tudor-Locke and colleagues [19] highlighted the need for more research studying the relationship between walking cadence and ambulatory intensity to establish a VPA step rate threshold. 

Another misgiving of this particular area of research, is that step rate thresholds have entirely focused on MVPA in regards to *absolute* intensity, commonly accepted as 3 metabolic equivalents (METs) for moderate intensity and 6 METS for vigorous-intensity respectively. The American College of Sport Medicine (ACSM) and the Canadian Society for Exercise Physiology (CSEP) recommends using individualized intensities for exercise prescription, where moderate is 40% of maximal aerobic fitness (VO_2max_) and vigorous is 60% VO_2max_ [20,21]. This establishes the need to identify step rate thresholds that are related to an individual’s fitness level and body type for the appropriate assessment and prescription of intensity-related physical activity. 

Therefore, the purpose of the current study was to identify step rates across a range of walking intensities in a broad sample of adults so that multi-level modelling of independent variables (i.e., METs, BMI, height, and/or leg length) could be used to calculate adults’ individualized step rate thresholds for absolute and relative MPA and VPA.

## 2. Materials and Methods

*Demographics*: A convenient sample of 43 adults (25 females) between the ages of 20–64 years (39.4 ± 15.2) volunteered to participate in this study. All participants were initially screened for age (18–65 years) and cleared for MVPA using the Physical Activity Readiness-Questionnaire Plus (PAR-Q+) [22]. Most participants (*n* = 24) answered “yes” to at least one question on the PAR-Q+. All participants completed a CSEP Physical Activity and Sedentary Behavior Questionnaire (PASB-Q) [21], a valid and reliable measure of weekly MVPA [23]. The study was approved by the Research Ethics Board at Acadia University (REB #15-20), and all subjects provided written informed consent before participating. 

*Anthropometrics*: Height and weight were measured without shoes using a calibrated stadiometer and scale (Health-O-Meter, McCook, IL, USA) to the nearest 0.5 cm and 0.1 kg, respectively. Leg length was measured using a tape measure (cm) as the distance from the greater trochanter to the floor without shoes and keeping their legs straight. This measure of leg length is similar to a previous report with the difference being that Beets and colleagues [16] measured participants with their footwear on.

*Aerobic Fitness*: Aerobic fitness was determined using the Ebbeling walking treadmill protocol [24]. The Ebbeling consists of 2, four-minute walking stages. The first stage is designed to reach a speed that elicits 60% of the participants’ estimated heart rate maximum (220 − age). The second stage consists of increasing the incline by 5% and maintaining the previously established speed. Treadmill speed and steady-state heart rate are used to estimate VO_2max_ [25]. A submaximal test was chosen over a maximal test to minimally influence subsequent walking assessment as it was most practical to complete fitness testing and step assessment in a single session. Furthermore, the time frame of this particular test (8 min) corresponds to the time restraints experienced by qualified exercise professionals to counsel, assess patients’ physical fitness and produce an optimal exercise program. Following the submaximal aerobic test, a resting period of 20–30 min was allotted to ensure participants returned back to a resting state.

*Treadmill Protocol*: Prior to testing, the metabolic cart (TrueOne 2400, Parvo Medics, Sandy, UT, USA) was calibrated using nitrogen and two primary standard gas mixtures to an error of 0.01%. The pneumotachometer was calibrated using a 3 L syringe that delivered fixed volumes at different flow rates. Volume calibration was verified to a value less than 0.1 L. Heart rate was monitored using a telemetry transmitter attached across the sternum (Polar, Lachine, QC, Canada). Participants were familiarized with the Borg scale, asking them to rate their ratings of perceived exertion (RPE) on a scale of 6–20 [26]. Participants were fitted with a headpiece, a two-way non-rebreathing valve (Hans-Rudolph, Inc., Shawnee, KS, USA), a nose-clip, and a mouth piece.

Participants performed up to six, 6-min walking bouts on a calibrated, level treadmill at 2.4, 3.2, 4.0, 5.6, 6.4, and 7.2 km/h (1.5, 2.0, 2.5, 3.5, 4.0, 4.5 miles/hour, respectively). Each bout was separated by a 4-min standing rest period, to ensure minimal drift of metabolism between stages. The order of treadmill bouts was progressive because of concerns that some participants would be unable to walk at the higher speeds due to limitations in fitness. Considering the relationship between step rate and metabolic activity is likely altered at jogging and running pace in comparison to walking, only walking was permitted.

Steps were manually counted by two researchers during minutes 2–3 and 4–5 of each stage to obtain the gold standard step counts per minute for each stage. A video camera filmed the feet of the participant in case the researchers recorded greater than 1 step difference during a stage. The steps counted during minutes 2–3 and 4–5 of each stage were averaged and multiplied by a factor of 6 to determine the number of steps for each six-minute stage. Of relevance, the steps counted during minutes 2–3 and 4–5 were always within 2 steps/minute. The test was terminated by completing the protocol of all six stages, volitional fatigue, or if the participant reached 85% of their estimated heart rate maximum or RPE was greater than 17 [25]. An appropriate cool-down was administered by the researcher while monitoring their heart rate recovery. 

VO_2_ for each 6-min walking bout was obtained using indirect calorimetry, where steady state was defined as a heart rate change of less than 5 beats per minute, consistent with previous research [18]. Average breath-by-breath VO_2_ and heart rate data were recorded at 15-s intervals for the duration of the protocol. Steady-state VO_2_ for each participant was recorded as an average of the last four minutes of each bout to limit the variability introduced by oxygen kinetics at the onset of exercise in each stage and corresponded to the time periods when steps per minute were counted. 

*Data Analysis*: Statistics were completed in R (versions 3.4.1, R Foundation for Statistical Computing, Vienna, Austria) and SPSS (IBM, SPSS Statistics for Mac, Version 23.0. IBM Corp., Armonk, NY, USA). Descriptive statistics are presented in the text as mean ± standard deviation, or proportion (%). Statistical significance was accepted as *p* < 0.05.

Participants’ relative predicted VO_2max_ was divided by 3.5 mL/kg/min to calculate their maximum METs [27]. For *absolute* intensity, MPA and VPA were classified as 3.00–5.99 METs and >6.00 METs, respectively. For *relative* intensity, MPA and VPA were classified as 40–59% MET_max_ and >60% MET_max_, respectively. For each stage, METs were calculated by dividing steady-state VO_2_ by 3.5 mL/kg/min. Step length (meters/step) was determined by dividing treadmill speed (meters/min) by step rate.

Multiple regression, mixed models, and receiver operating characteristic (ROC) curve analyses were used to differentiate step rate cut points for both MPA and VPA, respectively. The multiple regression approach was used to develop an equation that predicts step rate using metabolic activity and a combination of: BMI, height, and/or leg length. This statistical method was used in previous studies comparing step rate to intensity-related physical activity [15]. However, multiple data points from each individual were used in the current analysis, thus violating the assumption of data independence. Therefore, mixed effects modelling was used to overcome this limitation by incorporating random intercepts to account for the data-dependence structure. ROC curves were used to evaluate optimal step rate cut points that resulted in the highest sensitivity (true positives) and specificity (true negatives) for intensity-related physical activity as derived via Youden’s index. For ROC area under the curve (AUC) analysis, below 0.70 was considered poor, 0.70–0.80 considered fair, 0.80–0.90 considered good, and 0.90–1.00 considered excellent [28]. Median values of leg length, height, and relative METs were inserted into regression and multi-level modelling equations to predict step rate thresholds.

Subject-level plots of step rates as a function of METs indicated that the relationship between these variables was curvilinear. The model presented by Beets and colleagues [16], which used METs, METs^2^, BMI, leg length, and BMI×METs as predictors of step rate, was used as the starting model. Parameters were added or removed (i.e., height, METs^3^, etc.) according to relative goodness of fit and model complexity based on a priori knowledge regarding the relationship among the variables of interest and the objective of this study. Of the candidate models, relative Akaike information criterion scores (AIC; a model comparison measure) were used to determine which among them were most probable to minimize the information loss (i.e., which model was closer to the “true model” or the data-generating model). AIC comparisons were used to identify which models are best at trading-off bias versus variance among the fitted model parameters [29]. As such, AIC comparisons were used to identify which models were expected to maximize predictive accuracy and minimize predictive error. Model diagnostics were run for the best mixed model, for each height, BMI, and/or leg length as predictors. Assumptions of normality, homoscedasticity, and independence were assessed via residual plots. The relationship among predictors was assessed via scatterplots.

## 3. Results

The mean ± standard deviation (range) BMI, height, and leg length of the sample were 27.9 ± 6.1 (18–43) kg/m^2^, 1.71 ± 0.09 (1.55–1.90) meters, and 97.1 ± 6.9 (82–106) centimeters, respectively. Median values for BMI, height, leg length, and height were 26.6 kg/m^2^, 169.5 cm, and 98.0 cm, respectively. The sample had a predicted VO_2max_ of 41.2 ± 10.2 mL/kg/min and self-reported 216 ± 138 min of MVPA per week. All outcome variables progressively increased with faster walking speeds (see Table 1). Mean relative moderate METs (40% MET_max_) and relative vigorous METs (60% MET_max_) occurred at 4.7 and 7.1 METs, respectively in this sample population. Median relative moderate (40% MET_max_) and vigorous METs (60% MET_max_) were also 4.7 and 7.1 METs, respectively.

*Multiple Regression*: The multiple regression model generated to predict MPA and VPA step rates from METs, BMI, and leg length (cm) is presented in Supplement Table S1. The leg length and BMI regression model accurately predicted step rates (R^2^ = 0.807; *p* < 0.001). Predicted absolute and relative intensity related physical activity are presented in Table 2. Removing BMI from the regression model resulted in a similar fit to the model including BMI (Supplement Table S2). Furthermore, it predicted similar MVPA step rates as highlighted in Table 2, and resulted in an ~0.55 spm decrease in step rate for a 1 cm increase in leg length. The leg length regression model (R^2^ = 0.806; *p* < 0.0001) is presented in Supplement Table S1.

The multiple regression model generated to predict moderate and vigorous step rates from METs, BMI, and height (cm) is presented in Supplement Table S1. The model had an R^2^ = 0.829 (*p* < 0.0001), which was greater than the multiple regression including BMI and leg length (see Supplement Table S1). Furthermore, it predicted similar values to the BMI and leg length model (see Table 2). Additionally, removing BMI from the height regression model did not change its predictive capabilities (R^2^ = 0.828; *p* < 0.0001).

*Mixed Effects Modelling*: Similar to Marshall et al., (15), intercepts were allowed to vary among participants (i.e., random intercept modelling was used). The mixed effects model using leg length and BMI is presented in Supplement Table S1. According to AIC scores, when BMI terms (BMI, MET × BMI) were omitted from the model, the model was 2.7 times more probable to minimize the information loss than when these terms were included, consistent with the multiple regression analysis. Regardless, the non-BMI mixed effects model yielded similar predicted step rates (3.00 METs = 100.0 spm, 40% MET_max_ = 124.1 spm, 6.00 METs = 132.3 spm, and 60% MET_max_ = 137.5 spm) to the multi-level model including BMI (see Table 2). The model is presented below:

Step Rate = 41.300 − (0.585 × leg length) + (60.660 × METs) − (8.677 × METs^2^) + (0.448 × METs^3^)

**Figure 1:**
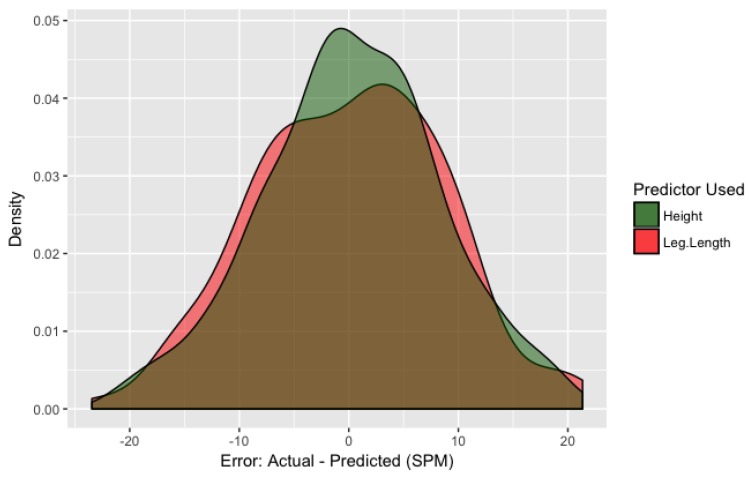
Density distribution of within-sample prediction error (spm) for both height (green shade) and leg length (red shade) models.

**Figure 2:**
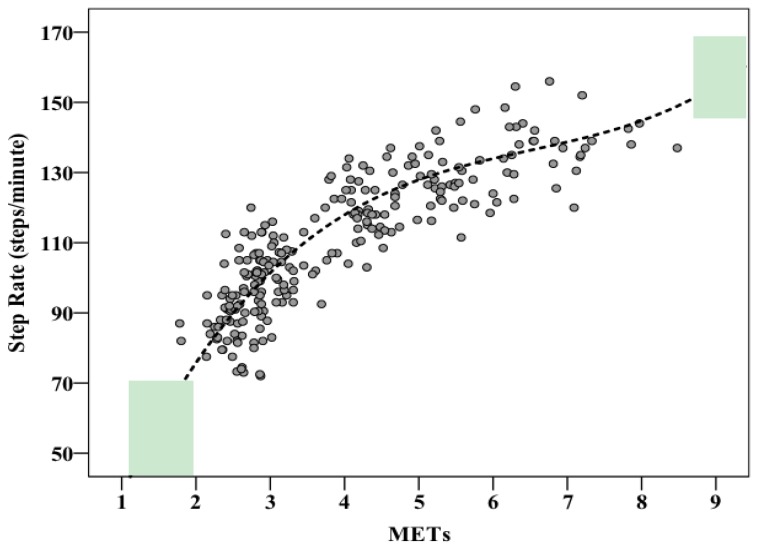
Scatterplot demonstrating the relationship between metabolic equivalents and step rate. The reference line is the mixed model including height using the sample’s median height (169.5 cm).

When comparing mixed models, the model that included height instead of leg length was over 21 times as probable to minimize the information loss. Figure 1 displays the distribution of within-sample prediction error for both leg length and height models. Error was measured as the difference between the equation predictions (using the fixed effect coefficients) and the actual participant-wise data points. It appears as though the bulk of the error falls between ±10 spm, with the largest errors nearing 15–20 spm. The average error for the height-adjusted model is 0.1 ± 8.1 spm. It can also be seen that the height model outperforms the leg length model as the distribution’s narrower. When leg length and height models both include BMI, the height model is over 18 times as probable to minimize the information loss. The mixed effects model including BMI and height is presented in Supplement Table S1. The most accurate model to predict step rate uses height (cm) and METs, and is presented in Figure 2 using the sample’s median height (169.5). The model is presented below:

Step Rate = 73.490 − (0.513 × height) + (59.867 × METs) − (8.500 × METs^2^) + (0.436 × METs^3^).

*Receiver Operating Characteristic Curves*: Data used for absolute MVPA and relative MVPA calculations were dependent upon the average MET values for the respective walking stages. The absolute moderate intensity physical activity (3 METs) ROC curve was generated based on stages 2–4 (mean ranging from: 2.8–4.3 METs). The optimal step rate was 106.8 spm, with 74% correctly classified as achieving moderate intensity and 81% correctly classified as not achieving moderate intensity. The AUC was 0.827 (SE = 0.04, *p* < 0.001, 95% CI = 0.757–0.898). The relative moderate intensity physical activity (4.72 METs) ROC curve was generated based on stages 3–5 (mean ranging from 3.2 to 5.3 METs). The optimal step rate was 120.0 spm, with 68% correctly classified as achieving moderate intensity and 66% correctly classified as not achieving moderate intensity. The AUC was 0.741 (SE = 0.04, *p* < 0.001, 95% CI = 0.655–0.826).

For both absolute and relative VPA (6.00 METs and 7.09 METs), the ROC curve was generated based on stages 5 and 6 (mean ranging from 5.3 to 6.8 METs). The optimal step rate for both was 134.3 spm. The sensitivity and specificity for absolute VPA was 77% and 78%, respectively, while relative VPA had sensitivity and specificity values of 56% and 59%. The AUC for absolute VPA was 0.805 (SE = 0.06, *p* < 0.001, 95% CI = 0.698–0.912). The AUC for relative VPA was insignificant at 0.572 (SE = 0.09, *p* = 0.39, 95% CI 0.404–0.739).

## 4. Discussion

The primary purpose of this current study was to identify step rates across a range of walking intensities so that mathematical modelling could be used to predict step rate thresholds for MPA and VPA using METs, BMI, height, and/or leg length as predictor variables. We observed that the mixed effects model that included MET, MET^2^, MET^3^, and height, but not BMI or leg length as predictor variables (see Supplement Table S1) most accurately predicted step rate in this heterogeneous sample of adults (average error 0.1 spm). In regards to traditional metabolic values that correspond to MPA (i.e., 3 METs), this study validates the 100 spm public message [15], with a step rate of 101 corresponding to 3 METs. For absolute VPA (i.e., 6 METs), the proposed model in this study estimated a step rate of ~134 spm, using a median value of height (169.5 cm). Furthermore, relative MPA (40% MET_max_; 4.7 METs) and VPA (60% predicted MET_max_; 7.1 METs) corresponded to step rate thresholds of ~125 spm and ~139 spm, respectively. 

The objective evidence behind the current public health message of 3000 steps in 30 min was severely understudied at the time of their inception [15]. However, despite the relatively small sample size the guidelines originated from (*n* = 147) [15,18], and lack of consideration for height and leg length, the recommendations appear to be valid based on our equations, which take such anthropometric factors into account. Of relevance, the BMI of participants in the Marshall et al. (15) study was 28 ± 4 kg/m^2^ and 30 ± 6 kg/m^2^ for men and women, respectively. Whereas BMI in the Tudor-Locke et al. (18) study was 25 ± 4.7 kg/m^2^ and 22 ± 2 kg/m^2^ for men and women, respectively. Altogether, to achieve the benefits of moderate intensity physical activity, a good “rule of thumb” is to recommend 3000 steps in 30 min, however it should be acknowledged that significantly taller or longer legged individuals will have to take less steps to achieve such an intensity. More specifically, for a 10 cm increase in height (i.e., 170 to 180 cm) the step rate threshold decreases by ~5 spm (5.13 spm). To further illustrate this point, for an individual who is 6 feet tall (182.9 cm) to achieve MPA and VPA intensity should be recommended to walk at 95 spm (3 METs) and 127 spm (6 METs), respectively, whereas an adult who is 5 feet tall (152.4 cm) should be recommended to walk at 110 spm and 143 spm for the same intensity; highlighting the potential for error in determining step rate thresholds when individualizing for height is not considered. Healthcare providers and exercise professionals may simply input their patients’ height and desired metabolic intensity to calculate an associated step rate threshold that would correspond to MPA or VPA. 

As mentioned, VPA corresponds to ~134 spm, which is higher compared to a previous study that related steps per minute to metabolic equivalents (6 METs = 125 spm) using a linear model in 19 healthy young adults [17]. Another study that specifically studied only MPA estimated 6 METs corresponding to 147–170 spm [14], which is unachievable for many individuals and likely drastically overestimated VPA. Both types of analysis and predictor variables used in this study (leg length or height) yielded similar values; interestingly, at a rate of 134 spm, or ~4000 steps in 30 min of physical activity. Therefore, future studies should assess the role of VPA-based pedometer based goals and evaluate the impact of vigorous exercise bouts (1350 steps versus 1000 steps in 10 min) on physiological outcomes.

Daily step count is a commonly used measure of physical activity level, but it does not provide information about physical activity intensity. Herein, stepping cadence may be used in free-living settings to assist individuals in meeting physical activity guidelines that are based on MVPA, not steps. The applications and limitations of using stepping cadence in free-living settings are reviewed in more detail by Tudor-Locke and Rowe [30]. To the authors’ knowledge, this is the first study to evaluate relative intensity MVPA step rate thresholds in a diverse sample of adults. Due to differences in aerobic fitness, calculating MPA and VPA based on a percentage of maximal aerobic fitness individualizes physical activity intensities. Individualizing physical activity intensities is recommended by CSEP and ACSM [20,21], and is particularly important for lesser fit individuals who have difficulty achieving MPA and VPA in absolute terms (i.e., 3 METs & 6 METs), and for very fit individuals who easily exceed absolute intensity thresholds. Although such step rates are variable due to the diverse degrees of aerobic fitness in our sample, the results show a considerably strong sensitivity and specificity (Area under curve: 0.741; *p* < 0.001) for a moderate-relative-intensity step rate of ~125 spm. However, given that the study’s reference equation is provided, it is recommended to individually tailor step rates when deemed appropriate (e.g., physical activity monitor studies), based on an individual’s personal level of fitness and calculated moderate and vigorous MET targets.

Limitations of this study are based on the fact that this assessment was a laboratory determination across a defined set of walking speeds in adults and therefore the equations identified apply to step rates between ~85–140 spm. Although a broad range of walking conditions was used, more stages, especially in the vigorous zone, might have improved the predictions relative to VPA. Particularly for average VPA in relative terms (60% MET_max_), as some participants were unable to walk at the cadence required to meet this intensity (7.1 METs). However, for VPA in absolute terms, 31 of the 43 participants (72%) reached 6 METs or higher by stage 5 or stage 6, which supports the validity of our prediction of absolute intensity VPA step rate thresholds. Likewise, there may be differences in applicability to free-living conditions, although evidence shows that walking on a treadmill and walking over ground are kinetically and kinematically equivalent [31] and elicit similar metabolic costs (i.e., within ~0.2 METs at slow, medium, and fast speeds in healthy individuals [32]. Other limitations were in the practicality of the design of the study; this study used a submaximal measure of aerobic fitness to determine fitness related relative intensities and assumed a constant (3.5 mL/kg/min) as an estimate of resting energy expenditure. The constant for resting energy expenditure is universally accepted as 3.5 mL/kg/min and is consistent with previous literature [19,27]. This is the first study of its kind to incorporate relative measures and given the practicality aspect of an 8-min aerobic test for practitioners in the field, the authors feel it was appropriate to create relative intensity points given the goal of the study to provide step rate thresholds for these professionals. The use of a sub-maximal assessment of aerobic fitness may be considered a limitation, however the single-stage treadmill protocol is a valid indicator of aerobic fitness [24]. As well, our sample size may be considered a limitation but it is similar to or greater than other similar research investigating MVPA step rate thresholds [16,17,18]. Each participant completed multiple exercise stages, resulting in a number of data points for each participant. Lastly, our results may not be extrapolated to older adults, highlighting that further research in this population is needed and should take into consideration relative intensity, leg length, and height. There is a need for a meta-analysis of multiple equations that predict step rate from metabolic values to strengthen step-based physical activity recommendations. Furthermore, research should evaluate the role of public health messages that include VPA (i.e., 4000 steps in 30 min) combined with pedometer-based goals in helping people lead more physically active lifestyles. 

## 5. Conclusions

A systematic review by Slaght and colleagues [33] suggests more evidence is required before prescribing walking cadence as a way of increasing physical activity in a practical setting, with the step rate thresholds associated with VPA being largely understudied [19]. With the adoption of walking as a common form of leisure-time physical activity, the information in this study provides support for the integration of physical activity monitoring and physical activity prescriptions by health and fitness professionals for the monitoring of accurate step rate thresholds associated with MVPA. It is appropriate for health care providers to recommend 3000 steps and 30 min for MPA, however, 4000 steps in 30 min appears to equate to VPA. Lastly, given the influence of aerobic fitness on classifications of moderate and vigorous exercise it is advised to use relative intensity (% of VO_2max_ or MET_max_) step rate thresholds.

## Figures and Tables

**Table 1:** Description of measured variables at each treadmill speed.

Stage (Speed)	Step Rate (spm)	METs	VO_2_ (mL/kg/min)	Step Length (m)	Heart Rate (bpm)	RPE (6–20)
Stage 1 ^a^(2.4 km/h)	85.5 ± 7.6	2.55 ± 0.39	8.93 ± 1.36	0.47 ± 0.04	88.9 ± 12.5	7.2 ± 1.5
Stage 2 ^b^(3.2 km/h)	97.3 ± 7.4	2.84 ± 0.37	9.93 ± 1.29	0.55 ± 0.04	90.1 ± 12.3	7.9 ± 2.0
Stage 3 ^c^(4.0 km/h)	106.0 ± 6.7	3.15 ± 0.41	11.01 ± 1.43	0.63 ± 0.04	91.6 ± 12.2	8.6 ± 2.2
Stage 4 ^d^(5.6 km/h)	120.2 ± 6.7	4.31 ± 0.43	15.07 ± 1.49	0.78 ± 0.04	105.5 ± 15.5	9.6 ± 2.7
Stage 5 ^e^(6.4 km/h)	128.0 ± 7.2	5.27 ± 0.38	18.00 ± 3.20	0.84 ± 0.05	116.0 ± 15.8	11.1 ± 2.2
Stage 6 ^f^(7.2 km/h)	138.5 ± 8.4	6.79 ± 0.65	21.06 ± 7.96	0.87 ± 0.05	132.2 ± 18.4	12.3 ± 3.3

Note: Data presented as mean ± SD; ^a^ 1.5 mph, *n* = 43; ^b^ 2.0 mph, *n* = 43; ^c^ 2.5 mph, *n* = 42; ^d^ 3.5 mph, *n* = 41; ^e^ 4.0 mph, *n* = 38; ^f^ 4.5 mph, *n* = 30; METs = Metabolic Equivalents (3.5 mL·kg^−1^·min^−1^); RPE = Ratings of Perceived Exertion (6–20).

**Table 2:** Minimum step rates for both absolute and relative moderate and vigorous intensity walking as established using multiple regression, mixed model, and ROC curve analyses.

Variable	Intensity Related Physical Activity Minimum Step Rates			
Analysis	Moderate Intensity (3 METs)	Relative Moderate Intensity (40% MET_max_)	Vigorous Intensity (6 METs)	Relative Vigorous Intensity (60% MET_max_)
Multiple Regression (Leg Length)	99.89	122.81	132.58	137.56
Mixed Model (Leg Length)	99.98	124.09	132.31	137.50
Multiple Regression (Height)	101.25	124.69	134.56	139.10
Mixed Model (Height)	101.41	125.59	133.91	139.11
ROC Curve	106.75	119.75	134.25	134.25

Note: Predicted VO_2max_ was divided by 3.5 mL/kg/min to calculate maximum metabolic equivalents (MET_max_). The median value of leg length was used in multiple regression analysis and mixed model analysis estimates of intensity related physical activity. Models including BMI are presented in Supplement Tables S1 and S2. BMI = body mass index (kg/m^2^). ROC = receiver operating characteristic.

## Supplementary Materials

The following are available online at http://www.mdpi.com/1660-4601/15/11/2454/s1, Table S1: Multiple regression and Mixed Effects Modelling equations to predicted step rate, Table S2: Intensity-related physical activity minimum step rates across different types of analysis.
