# Supplementary material for: Step Rate Thresholds Associated with Moderate and Vigorous Physical Activity in Adults
Source: Int J Environ Res Public Health. 2018 Nov 3;15(11):2454. doi: 10.3390/ijerph15112454 (PMC6266480; doi:10.3390/ijerph15112454)
Supplement: Supplementary file 1 [file ijerph-15-02454-s001.docx]

Supplement Materials

**Table S1.** Multiple regression and Mixed Effects Modelling equations to predicted step rate.

| **Type of Analysis** | **Variables** | **R^2^ (*p*-Value)** | **Equation** |
| --- | --- | --- | --- |
| Multiple Regression |  |  |  |
|  | METs, BMI (kg/m^2^) and Leg Length (cm) | R^2^ = 0.807 (*p* < 0.0001) | 60.173 − (0.543 × leg length) + (41.448 × METs) − (4.210 × METs^2^) + (0.166 × METs^3^) + (0.422 × BMI) − (0.116 × BMI × METs) |
|  | METs, BMI (kg/m^2^) and Height (cm) | R^2^ = 0.829 (*p* < 0.0001) | 101.497 − (0.511 × height) + (37.684 × METs) − (3.543 × METs^2^) + (0.114 × METs^3^) + (0.301 × BMI) − (0.076 × BMI × METs) |
|  | METs and Leg Length (cm) | R^2^ = 0.806 (*p* < 0.0001) | 73.023 − (0.559 × leg length) + (38.337 × METs) − (4.207 × METs^2^) + (0.165 × METs^3^) |
|  | METs and Height (cm) | R^2^ = 0.828 (*p* < 0.0001) | 110.362− (0.517 × height) + (35.781 × METs) − (3.546 × METs^2^) + (0.115 × METs^3^) |
| Mixed Effects |  |  |  |
|  | METs, BMI (kg/m^2^) and Leg Length (cm) |  | 33.023 − (0.573 × leg length) + (62.681 × METs) − (8.686 × METs^2^) + (0.449 × METs^3^) + (0.268 × BMI) − (0.076 × BMI × METs) |
|  | METs, BMI (kg/m^2^) and Height (cm) |  | 65.088 − (0.499 × height) + (61.792 × METs) − (8.523 × METs^2^) + (0.438 × METs^3^) + (0.217 × BMI) − (0.069 × BMI × METs) |
|  | METs and Leg Length (cm)* |  | 41.300 − (0.585 × leg length) + (60.660 × METs) − (8.677 × METs^2^) + (0.448 × METs^3^) |
|  | METs and Height (cm)* |  | 73.490 − (0.513 × height) + (59.867 × METs) − (8.500 × METs^2^) + (0.436 × METs^3^) |

* = Most accurate models to predict step rates using metabolic equivalents as the primary independent variable.

**Table S2.** Intensity-related physical activity minimum step rates across different types of analysis.

|  | | **Intensity Related Physical Activity Step Rates** | | | |
| --- | --- | --- | --- | --- | --- |
| **Analysis** | **Variables** | **Moderate Intensity**  **(3 METs)** | **Relative Moderate Intensity**  **(40% MET_max_)** | **Vigorous Intensity**  **(6 METs)** | **Relative Vigorous Intensity**  **(60% MET_max_)** |
| Multiple Regression | Leg Length & BMI | 99.88 | 122.93 | 132.67 | 137.73 |
|  | Leg Length | 99.89 | 122.81 | 132.58 | 137.56 |
|  | Height & BMI | 101.02 | 124.22 | 133.88 | 138.19 |
|  | Height | 101.25 | 124.69 | 134.56 | 139.10 |
| Mixed Effects | Leg Length & BMI | 99.97 | 124.08 | 132.32 | 137.55 |
|  | Leg Length | 99.98 | 124.09 | 132.31 | 137.50 |
|  | Height & BMI | 101.31 | 125.47 | 133.79 | 139.00 |
|  | Height | 101.41 | 125.59 | 133.91 | 139.11 |
